# Acquired Antibiotic Resistance in Escherichia coli Exposed to Simulated Microgravity: Possible Role of Other Space Stressors and Adaptive Responses

**DOI:** 10.1128/mBio.00165-19

**Published:** 2019-03-26

**Authors:** S. M. J. Mortazavi

**Affiliations:** a Diagnostic Imaging Department, Fox Chase Cancer Center, Philadelphia, Pennsylvania, USA

**Keywords:** antibiotic resistance, microgravity, radiation, space

## LETTER

I read with interest the paper recently published by Tirumalai et al. entitled “Evaluation of acquired antibiotic resistance in Escherichia coli exposed to long-term low-shear modeled microgravity and background antibiotic exposure” (1). That paper addresses the effect of low-shear modeled microgravity (LSMMG) conditions with respect to altering antibiotic stress resistance in microbial ecosystems. Tirumalai et al. state that during a long-term space mission, exposure to a wide range of stress factors such as microgravity can lead to immune suppression that increases the infection risk as follows. “Stress factors experienced during space include microgravity, sleep deprivation, radiation, isolation, and microbial contamination, all of which can promote immune suppression (1, 2). Under these conditions, the risk of infection from opportunistic pathogens increases significantly, particularly during long-term missions (3). If infection occurs, it is important that the infectious agent should not be antibiotic resistant. Minimizing the occurrence of antibiotic resistance is, therefore, highly desirable.” Although the paper authored by Tirumalai et al. addresses a very challenging issue and can be considered a significant contribution in this field, it has at least one shortcoming. This shortcoming comes from not considering the key role of space radiation in acquired antibiotic resistance and the complex interactions among space radiation and microgravity. The possible interplay of space radiation and microgravity in DNA damage and responses to DNA damage has been addressed recently (2).

My colleagues and I have previously shown that exposure to both ionizing (3) and nonionizing (4) electromagnetic radiation can make bacteria more resistant to antibiotics (the so-called “bacterial adaptive response”). The adaptive response can be defined as increased resistance in living organisms preexposed to a low-level stressor (e.g., low doses of ionizing radiation) before exposure to a highly challenging stressor (5). Moreover, Mortazavi et al. have previously introduced the idea of the use of adaptive responses as an effective strategy for reducing the radiation risk in long-term manned space missions (6). A National Aeronautics and Space Administration (NASA) report entitled “Evidence report: risk of radiation carcinogenesis” (7), which was approved for public release on 7 April 2016, confirms the importance of studies of adaptive response in deep space missions. Given this consideration, not only bacteria but also astronauts can show adaptive responses in space. Therefore, during a space mission, acquired antibiotic resistance can be due to the complex interactions among key stress factors such as space radiation and microgravity.

In summary, although the paper by Tirumalai et al. addresses an important topic, its conclusions must be evaluated in terms of the issues noted in this letter. Extrapolating the results obtained in the very specific environment addressed in that study to other environments is not likely to yield credible results in view of the issues summarized in my comments.
